# Prevention and Management of Preterm Birth

**DOI:** 10.1155/2012/610364

**Published:** 2012-03-18

**Authors:** Yves Jacquemyn, Ronnie Lamont, Jérôme Cornette, Hanns Helmer

**Affiliations:** ^1^Antwerp University Hospital (UZA), 2650 Edegem, Belgium; ^2^Department of Obstetrics and Gynecology, Northwick Park Hospital, London, UK; ^3^Department of Obstetrics and Gynecology, Erasmus MC, University Medical Center, Rotterdam, The Netherlands; ^4^Department of Obstetrics and Gynecology, Vienna General Hospital, Medical University of Vienna, Vienna, Austria

Prevention and treatment of preterm delivery is not one of the success stories of modern medicine, preterm birth constitutes the major determinant of perinatal mortality and morbidity, and the long-term results of being born too early often lead to a shorter, less healthy life and a more difficult school and professional career. Different methods have been introduced to predict the advent of preterm labour in asymptomatic women, including fetal fibronectin and transvaginal ultrasound cervical length measurement. R. Arisoy and M. Yayla present data on the evaluation of the cervix in asymptomatic singleton pregnancies; they also address the most frustrating issue: what measures to take once a short cervix has been detected. They restrict their study to singleton pregnancies; although both cervical length and fetal fibronectin are good predictors of preterm delivery in twins, no intervention has proven useful in twins: vaginal progesteron makes no difference and cerclage even worsens the outcome.

Possibilities for real primary prevention are rare and include treatment of asymptomatic bacteriuria and periodontal disease. O. Huck et al. elaborate this last issue and present an excellent overview on both epidemiologic and pathophysiologic data. Another method proposed for primary prevention of preterm birth is the use of progesteron, including vaginal progesteron and systemic 17-hydroxyprogesterone caproate. Starting progesterone treatment can be based not only on cervical length or vaginal fibronectin but also on past obstetrical history. C. E. Ransom et al. comment on the use of 17-hydroxyprogesterone caproate and the influence of obstetric history.

 Some newer methods are on the border of being introduced to clinical practice; one such candidate is near-infrared spectroscopy. K. M. Power and colleagues present the use of near-infrared spectroscopy of amniotic fluid to assess preterm delivery.

Once preterm labour has been established, tocolytics are (all too) often used, and what their exact place in treatment is remains open for discussion. Hubinont and F. Debieve present a concise update on tocolysis. In case preterm delivery seems unavoidable, the optimal mode of fetal monitoring and the mode of delivery have to be chosen. Fetal heart rate monitoring in the preterm period constitutes a special challenge and is further commented by K. Afors and E. Chandraharan, while S. R. Bhatta and C. R. Keriakos discuss the optimal way of delivering the preterm baby in vertex position.

As the articles in this issue demonstrate, preterm labour and delivery constitute one of the major challenges of obstetrics in the 21st century.

*Yves Jacquemyn* *Yves Jacquemyn* *Ronnie Lamont* *Ronnie Lamont* *Jérôme Cornette* *Jérôme Cornette* *Hanns Helmer* *Hanns Helmer*

